# Bittersweet emotion and narrative transportation in streaming drama: cognitive and emotional routes to persuasion

**DOI:** 10.3389/fpsyg.2026.1815433

**Published:** 2026-06-12

**Authors:** Hark Shin Kim, Jongsoo Lim

**Affiliations:** 1 Department of Media and Communication, Sejong University, Seoul, Republic of Korea; 2 Department of Media and Communication, Institute of Platform-Media, Sejong University, Seoul, Republic of Korea

**Keywords:** behavioral intention, bittersweet emotion, emotion and cognition, moral belief formation, narrative transportation

## Abstract

This study examines how cognitive and emotional components of the Extended Transportation-Imagery Model (ETIM) are associated with relational moral beliefs and behavioral intentions in the context of serialized streaming drama. Survey data from 300 Korean viewers of a Netflix original series were analyzed using structural equation modeling. Character identification, perceived verisimilitude, and cultural familiarity were positively associated with narrative transportation. Narrative transportation was, in turn, associated with relational moral beliefs both directly and indirectly through bittersweet emotion. Bittersweet emotion, characterized by the coexistence of warmth- and sadness-related affect, was associated with reflective engagement with relational moral beliefs and related behavioral intentions. Rather than making causal claims, this study provides cross-sectional evidence consistent with a dual-route interpretation of narrative engagement in long-form streaming contexts. These findings highlight the possible relevance of emotionally complex storytelling for understanding belief-oriented responses to serialized narratives.

## 1 Introduction

Streaming dramas driven by Netflix have become one of the most dominant narrative formats in contemporary media consumption. Unlike short narrative stimuli commonly used in experimental research, serialized streaming dramas are characterized by sustained and repeated exposure across multiple episodes. This format keeps viewers in contact with the same characters and story world across multiple episodes, making it a relevant context for examining how narrative engagement may be associated with emotional and interpretive responses over an extended viewing experience.

Despite the prominence of narrative transportation as a key mechanism of narrative persuasion, much of the existing literature has focused on brief and time-limited narrative exposure (Green and Brock, 2000; Green and Appel, 2024). Consequently, less is known about how transportation operates in long-form streaming narratives, where cognitive and emotional processes may unfold cumulatively rather than instantaneously.

To address this gap, the present study uses the Extended Transportation-Imagery Model (ETIM) as a conceptual framework for examining associative relationships in a long-form streaming context. Focusing on a Netflix original series, the study examines whether narrative transportation, bittersweet emotion, and relational moral beliefs are associated with relational moral behavioral intentions. In doing so, it explores whether a pattern consistent with narrative persuasion research can also be observed in a more ecologically realistic streaming environment.

### 1.1 Narrative transportation in serialized streaming

Narrative transportation refers to a psychological state in which individuals become cognitively and emotionally absorbed in a story world, with attention temporarily shifted away from immediate surroundings (Gerrig, 1993; Green and Brock, 2000). For example, rather than reducing attention overall, narrative transportation redirects attention away from immediate surroundings and toward the fictional world presented in the story. In this state, individuals mentally simulate events, follow narrative relations, and experience characters’ emotions in a way that may be associated with shifts in interpretation and evaluative responses, often through experiential processing rather than explicit argumentation (Green and Appel, 2024; Green and Brock, 2000).

Most empirical research on transportation has relied on short, time-limited narrative exposure, typically limited to one-time exposures such as short stories or public service messages (Gerrig, 1993; Green and Brock, 2000). Furthermore, a systematic literature review found that narrative transportation studies have primarily focused on marketing-generated stories like advertisements and short brand narratives, rather than long-form serialized content (Thomas and Grigsby, 2024). However, serialized streaming dramas differ fundamentally in structure. Rather than offering a single, bounded episode of immersion, they engage individuals with recurring characters and evolving moral dilemmas over multiple episodes, fostering sustained engagement. In such contexts, transportation may be experienced in relation to an unfolding narrative encountered across multiple episodes rather than within a single brief exposure. Viewers return to the same narrative world across episodes, reinterpreting earlier events in light of developments and deepening emotional investment over time (Warren, 2020; Erickson et al., 2019).

To account for these dynamics, this study employs the ETIM as an integrative framework for examining how narrative features are associated with transportation and how transportation in turn, relates to downstream belief- and intention-related variables (Van Laer et al., 2014). ETIM conceptualizes narrative persuasion as involving identifiable antecedents and interrelated cognitive and emotional processes. While the model has been widely applied to advertising and short narrative formats, its application to long-form serialized streaming remains comparatively limited. Applying ETIM to this context allows us to examine whether sustained immersion is associated with relational moral beliefs and relational moral behavioral intentions in ecologically realistic viewing conditions.

### 1.2 Narrative context and moral orientation of the series

To examine narrative transportation in a sustained streaming context, this study centers on Netflix original series, *When Life Gives You Tangerines* (released in March 2025). Set in Jeju Island during the mid-twentieth century, the narrative depicts everyday relational choices that gradually move away from rigid role-based hierarchy and toward interaction patterns characterized by empathy, reciprocity, and mutual regard. Rather than presenting overt ideological confrontation, the series portrays subtle adjustments in how individuals respond to one another in ordinary social life.

In this study, relational moral beliefs refer to viewers’ endorsement of relational norms represented in the narrative world, particularly those concerning empathy, relational equality, and reciprocal care. By contrast, relational moral behavioral intentions refer to viewers’ expressed readiness to translate such endorsed norms into their own anticipated interpersonal conduct.

This relational moral orientation reflects movement toward an ethic grounded in mutual responsiveness and shared consideration. The narrative does not frame this movement as dramatic social transformation. Instead, it presents incremental changes in relational expectations that emerge through repeated interpersonal encounters. Such gradual changes in how characters treat one another provide a useful context for examining whether viewers become more supportive of empathy, reciprocity, and relational equality.

Importantly, these relational dynamics unfold within an emotional atmosphere marked by the simultaneous presence of warmth and quiet sorrow. The series does not rely on uniformly positive or negative affect but instead evokes emotionally mixed responses that invite reflective engagement. This bittersweet emotional texture is theoretically relevant because it may keep viewers engaged while prompting them to reconsider how characters respond to loss, care, and responsibility. By allowing viewers to acknowledge constraint, loss, and attachment at the same time, the narrative creates conditions in which alternative relational possibilities can be imagined rather than immediately resisted. In this sense, bittersweet emotional experience functions as a psychological context that supports reflective engagement with evolving relational norms.

Across the series, these relational orientations are not presented as abstract values but as recurring interpersonal situations embedded in family and community life. The narrative repeatedly depicts moments in which characters confront unequal expectations, endure relational loss, offer care under constraint, and renegotiate their responsibilities toward others. Such scenes do not function as isolated moral lessons. Rather, they gradually build a pattern in which fairness, reciprocity, and mutual regard become salient through everyday interaction. This scene structure is also relevant to the present study’s key constructs: the recurring tension between care and loss provides a concrete narrative basis for bittersweet emotional responses, while the repeated depiction of relational negotiation provides a basis for examining viewers’ endorsement of relational moral beliefs.

### 1.3 ETIM and antecedents of transportation

ETIM conceptualizes transportation as a process structured by antecedent conditions and related cognitive and emotional responses within narrative experiences (Van Laer et al., 2014). In the present study, the model is applied as a conceptual framework for examining transportation in a serialized streaming context. Within this adaptation, three antecedent conditions are treated as especially relevant for long-form drama viewing: character identification, perceived verisimilitude, and cultural familiarity. While character identification and perceived verisimilitude reflect more established transportation-related conditions, cultural familiarity is retained here as a context-specific antecedent that may facilitate entry into the narrative world in culturally embedded streaming narratives.

Character identification refers to the extent to which viewers adopt the character’s perspective, feel psychological intimacy, and experience the narrative through the character’s goals and emotions (Cohen, 2001). Specifically, identification directs viewers’ attention and imagery to a stable focus (e.g., primary protagonist), thereby facilitating immersion, reducing psychological distance, and enhancing experiential engagement. In long-form narratives, repeated encounters with the same characters may make character identification especially relevant to transportation (Erickson et al., 2019; Warren, 2020).

Perceived verisimilitude enables viewers to imagine narrative events as if they were real experiences and immerse themselves in them. This refers to the psychological plausibility and internal consistency of the narrative world and is distinct from objective factual accuracy (Busselle and Bilandzic, 2008, 2009). Verisimilitude facilitates sustained transportation when narratives present consistent character motivations, believable causal relationships, and credible social contexts.

In the present study, familiarity is specified more concretely as cultural familiarity, referring to the extent to which viewers recognize and can readily interpret the social cues, values, customs, and everyday interactional patterns represented in the drama (Van Laer et al., 2014). This specification reflects the present research context, in which transportation may be supported not only by narrative plausibility and character engagement but also by the accessibility of culturally embedded meanings. Cultural familiarity is therefore retained as an antecedent of narrative transportation, while being treated as a contextual facilitative condition rather than as a standalone persuasive mechanism. In serialized streaming contexts, such familiarity may ease entry into the narrative world, whereas identification and perceived verisimilitude may be more central to sustained engagement over time (Van Laer et al., 2014; Erickson et al., 2019).

*H1a:* Character identification will be positively associated with narrative transportation in serialized streaming drama.

*H1b:* Perceived verisimilitude (realism) will be positively associated with narrative transportation in serialized streaming drama.

*H1c:* Cultural familiarity will be positively associated with narrative transportation in serialized streaming drama.

### 1.4 Transportation and bittersweet emotion

In the ETIM, the combination of immersive imagery simulation and empathy manifests emotional engagement, which is theorized to contribute to the persuasive power of narrative transportation (Van Laer et al., 2014). Building on Green and Brock’s (2000) original transportation-imagery model, ETIM reconceptualizes transportation, as a systematic process that combines understanding, imagery, and empathy. Cohesive, realistic, and personally relevant narratives prompt audiences to engage in this imagery-based simulation, allowing them to imagine and empathize with events as if they were directly involved in the story (Busselle and Bilandzic, 2009; Slater and Rouner, 2002). In this sense, emotional engagement is not merely incidental to transportation but may accompany its reflective and persuasive potential (Green and Appel, 2024).

In line with this perspective, recent work suggests that the emotional consequences of transportation are not dichotomous but multi-layered and ambivalent. Exploring the role of complex emotions in narrative persuasion beyond a focus on simple pleasure and empathy, Oliver and Hartmann (2010) proposed a pattern of bittersweet resonance, suggesting that the fusion of warmth and sadness may encourage reflective engagement with narrative meaning. Newman and Sachs (2023) similarly argue that bittersweet nostalgia, which is a combination of tenderness, loss, and longing, transcends hedonic escape and supports reflective self-evaluation. In the present drama context, such emotionally mixed responses are likely to be associated with recurring scenes in which affection, sacrifice, disappointment, and relational endurance are experienced together rather than separately.

In the present study, bittersweet emotion is treated as a specific form of ambivalent affect rather than as a broad label for all meaningful emotional responses. Ambivalent affective states have been described as experiences that contain both positive and negative aspects, and bittersweetness has been discussed as one such state in which positive and negative feeling tones may be integrated within a single experiential episode (Vaccaro et al., 2020). This understanding is also consistent with evidence that bittersweet scenes can elicit simultaneous feelings of happiness and sadness, rather than merely alternating between the two over time (Larsen and Green, 2013). In serialized streaming drama, such mixed feelings may arise across recurring encounters with care, loss, humor, endurance, and relational change.

In serialized streaming dramas, emotionally mixed responses may be observed in relation to recurring encounters with care, loss, humor, endurance, and relational change. Recent research suggests that immersive story processing is often accompanied by emotional shifts, and that such shifts are positively associated with transportation and with narrative impact-related outcomes (Winkler et al., 2023). In this sense, bittersweet emotion in the present study is treated not as a separate persuasive mechanism in itself, but as a more specific affective response that may accompany transportation and support reflective engagement with narrative meaning (Green and Appel, 2024).

Accordingly, this study conceptualizes bittersweet emotion as a specific affective response that may accompany transportation in long-form streaming drama. In this context, stronger transportation may be associated with emotionally mixed responses that integrate compassion with reflective sadness. Based on this reasoning, the following hypothesis is proposed:

*H2:* Narrative transportation will be positively associated with bittersweet emotion.

### 1.5 Narrative transportation and relational moral beliefs: the cognitive route

The ETIM proposes that narrative persuasion may involve parallel emotional and cognitive processes (Van Laer et al., 2014). Within this framework, narrative transportation encompasses cognitive activities, such as sustained attention, narrative comprehension, and imagery-based simulation, which may be associated with belief-oriented processing (Busselle and Bilandzic, 2009; Kuijpers et al., 2017). Audiences may construct the story world by interpreting character motivations, moral tensions, and relational outcomes, and these interpretive processes may correspond with endorsement of relational norms represented in the narrative (Green, 2004; Green and Appel, 2024).

Cognitive transportation functions like a narrative simulation, immersing audiences in an experience beyond everyday cognition. What Gerrig (1993) described as narrative journeys allows viewers to transfer their perspectives into the fictional world, rehearsing social scenarios, predicting outcomes, and exploring moral dilemmas. Green (2021) argues that these simulations enable moral reasoning even without explicit argumentation. Likewise, Mar and Oatley (2008) show how such simulations facilitate social learning through imagined perspective-taking and ethical judgments in interpersonal contexts. In the present narrative context, this kind of cognitive engagement may remain salient as viewers follow how characters interpret obligations, fairness, and reciprocity across evolving family and community relationships.

Crucially, this cognitive aspect of transportation may be associated with belief-oriented evaluation even in the absence of intense emotional arousal. As viewers follow a coherent narrative logic, they may come to endorse relational norms such as fairness, responsibility, and relational equality. Slater and Rouner (2002) suggest that, when audiences are absorbed in a narrative, resistance to persuasive content may be reduced because attention is directed toward the unfolding story rather than toward generating counterargument. In serialized dramas, these cognitive processes may be reinforced through repeated exposure across episodes.

As audiences follow a coherent storyline and mentally organize its relational logic, they may engage in evaluative reflection on the relational norms represented in the narrative. In this sense, belief-oriented evaluation may arise from sustained cognitive engagement with story structure and meaning, even in the absence of intense emotional arousal. Bilandzic and Busselle (2013) highlight how narrative engagement may prompt audiences to reinterpret story events, while Kuijpers et al. (2017) describe sustained absorption as involving cognitive integration of narrative information across unfolding events.

From this perspective, relational moral beliefs are treated in the present study as a belief-oriented correlate of narrative transportation rather than as a directly observed product of belief change during exposure. More specifically, the construct refers to consciously reported evaluative orientations that prioritize empathy, fairness, and relational equality in interpersonal contexts. Emotional responses may accompany this pattern, but the current cross-sectional design does not permit firm conclusions about temporal ordering. Accordingly, the present study examines whether narrative transportation is positively associated with relational moral beliefs, independent of bittersweet emotional responses.

*H3:* Narrative transportation will be positively associated with relational moral beliefs, independent of bittersweet emotion.

### 1.6 Bittersweet emotion and relational moral beliefs: the emotional route

Whereas the cognitive aspects of narrative transportation may support belief-oriented processing through attention and comprehension, emotionally complex responses may constitute a parallel affective pathway that contributes to reflective meaning-making. Recent research suggests that meaningful narrative experiences often evoke multilayered emotional reactions that cannot be adequately captured by discrete affective categories such as joy or sadness alone (Green and Appel, 2024). In particular, the coexistence of warmth and sorrow has been discussed as an emotionally mixed state that may encourage reflective engagement with narrative meaning beyond hedonic enjoyment (Oliver and Hartmann, 2010; Newman and Sachs, 2023).

Within the context of serialized streaming narratives, such emotionally mixed responses may emerge across recurring encounters with themes of care, loss, reconciliation, and interpersonal responsibility. Hanich et al. (2014) describe these experiences as involving a simultaneous sense of tenderness and sadness that accompanies meaningful esthetic engagement. This is particularly plausible in the present drama context, where scenes of care, separation, endurance, and reconciliation are interwoven across episodes, creating conditions for emotional responses that are at once tender and sorrowful. Rather than functioning solely as momentary affective reactions, these emotional responses may facilitate evaluative reflection on interpersonal norms depicted in the narrative world.

From this perspective, bittersweet emotional experience may provide an affective context in which viewers consider relational dynamics such as empathy, reciprocity, and fairness. In this study, bittersweet emotion is treated not as emotional engagement in general, but as a more specific affective response that may accompany transportation in long-form narrative experience. Its relevance lies in the possibility that viewers remain open to relational meanings because warmth and sorrow are experienced together and reflectively processed (Vaccaro et al., 2020; Larsen and Green, 2013). More broadly, such mixed affect may also emerge in relational contexts marked by simultaneous attachment and loss (Dunbar, 2008). This interpretation is consistent with ETIM’s broader assumption that emotional engagement contributes to downstream interpretive processing within narrative persuasion (Van Laer et al., 2014). Accordingly, this study treats bittersweet emotion as an affective correlate through which narrative transportation may be indirectly associated with relational moral beliefs.

*H4:* Bittersweet emotion will be positively associated with relational moral beliefs.

*H5:* Narrative transportation will show an indirect association with relational moral beliefs via bittersweet emotion.

### 1.7 From relational moral beliefs to behavioral intentions

Following the cognitive and emotional processes through which narrative transportation may be associated with relational moral beliefs, behavioral intention represents the stage at which such belief-oriented processing may extend beyond the story world into everyday interaction. In the present study, relational moral beliefs and relational moral behavioral intentions are treated as related but conceptually distinct outcomes. Relational moral beliefs refer to evaluative endorsement of relational norms such as empathy, fairness, and mutual respect, whereas relational moral behavioral intentions refer to self-reported readiness to enact those endorsed norms in daily interaction. Prior research in persuasion and social cognition has consistently shown that internalized beliefs function as proximal determinants of individuals’ readiness to act in everyday contexts (Ajzen, 2020; McEachan et al., 2011). Within narrative persuasion research, beliefs shaped through engagement with story content may therefore be associated with subsequent intentions related to interpersonal conduct (Moyer-Gusé, 2008; Murphy et al., 2013). Merry and Payne (2025) similarly found that transportation in environmental narratives was associated with value-based beliefs and future-oriented intentions. Prior research has also suggested that narrative transportation may be associated with post-exposure communicative intentions, such as willingness to share or discuss narrative content (Wang and Tang, 2021).

In the context of serialized streaming narratives, repeated exposure to relational dilemmas and everyday moral choices may be associated with stronger endorsement of relational moral beliefs emphasizing empathy, reciprocity, and fairness. These beliefs, in turn, may be associated with behavioral intentions aligned with relational ethics, such as cooperative communication, empathic listening, or willingness to maintain social ties. Rather than depicting dramatic or heroic acts, streaming dramas often model routine interpersonal responses that may become cognitively or affectively salient through sustained narrative engagement (Green, 2021).

From this perspective, relational moral beliefs represent a belief-oriented mechanism through which narrative transportation may be associated with relational moral behavioral intentions. Accordingly, the following hypotheses are proposed:

*H6:* Relational moral beliefs will be positively associated with relational moral behavioral intentions.

*H7:* Narrative transportation will show an indirect association with relational moral behavioral intentions via relational moral beliefs.

*H8:* Narrative transportation will show an indirect association with relational moral behavioral intentions via bittersweet emotion and relational moral beliefs.

### 1.8 Integrative conceptual model

**Figure 1:**
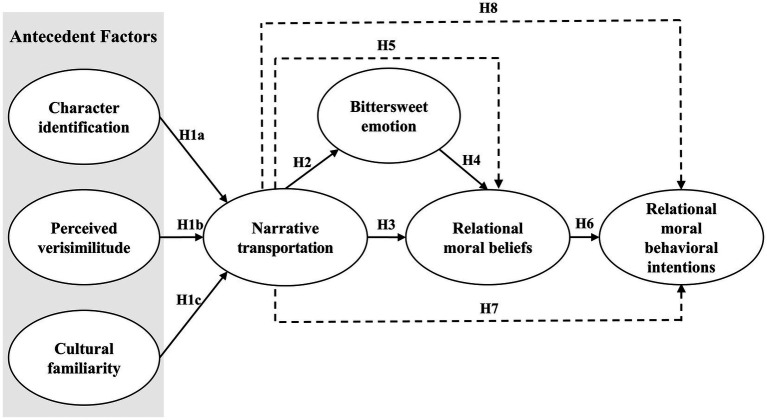
Conceptual framework of the study.

Building on the preceding theoretical discussions, this study proposes a dual-route framework of narrative transportation within serialized streaming contexts. As illustrated in Figure 1, the model combines antecedent, cognitive, emotional, and belief-related components derived from the ETIM (Van Laer et al., 2014) and subsequent elaborations on experiential and affective engagement in narrative persuasion (Green and Appel, 2024). The proposed model is not intended to establish a causal sequence but to examine whether a pattern consistent with dual cognitive and emotional pathways can be observed in cross-sectional survey data from viewers of a serialized streaming drama.

Three narrative antecedents, including character identification, perceived verisimilitude, and cultural familiarity, are expected to facilitate narrative transportation (H1a, H1b, H1c). As transportation intensifies, viewers may experience emotionally mixed responses characterized by the coexistence of warmth and subdued sorrow, conceptualized in this study as bittersweet emotion (H2). Such emotional responses may provide an affective context through which transportation becomes associated with relational moral beliefs (H4, H5), while transportation itself may also relate to these beliefs through cognitive engagement (H3). These belief-oriented outcomes, in turn, may be associated with relational moral behavioral intentions that reflect interpersonal orientations such as empathy, reciprocity, and cooperative communication (H6, H7, H8).

Applied to the narrative context of *When Life Gives You Tangerines*, this framework outlines how emotionally complex serialized storytelling provides a context in which viewers’ interpretations of relational norms may be examined alongside cognitive and affective aspects of narrative engagement. In this way, streaming narratives are conceptualized not merely as entertainment content but as experiential environments in which viewers interpret and evaluate interpersonal values across repeated narrative exposure.

## 2 Materials and methods

### 2.1 Study design and data collection

Data were collected through an online survey conducted in August 2025 with 300 adult participants in South Korea, aged between 18 and 70. Respondents were recruited via M-Brain, a professional research firm that manages a nationwide research panel stratified by age, gender, and region. To be eligible, participants were required to reside in South Korea, be at least 18 years old, and have watched the entire Netflix series, *When Life Gives You Tangerines*. To confirm prior exposure to the series, participants first completed screening questions regarding their general Netflix use and their specific viewing experience with the series. Responses to these items indicated whether participants had watched the full series and were able to recall key narrative elements; only those meeting these criteria were allowed to proceed. Because the study relied on a post-viewing, cross-sectional survey of respondents who had completed the series before participation, the design is suited to examining associations among recalled narrative experiences, emotional responses, and belief- and intention-related variables rather than establishing causal or temporal order. As screening and response validation were completed before analysis, no additional researcher-level treatment of missing data was required, and SEM was estimated in AMOS using maximum likelihood.

The sample size was determined with consideration of the planned confirmatory factor analysis (CFA)/structural equation model (SEM) analysis and recruitment feasibility among viewers who had completed the full series. In this case, the sample size requirements for SEM are not based on a single universal cutoff but vary depending on the model characteristics (Wolf et al., 2013). Thus, the final sample of 300 respondents was considered adequate for the present model, which involved 60 estimated parameters.

### 2.2 Participants and sample characteristics

A total of 300 valid responses were obtained. The sample included 99 men (33.0%) and 201 women (67.0%). Ages ranged from 18 to 70, with respondents evenly distributed across four age groups: 20s or younger (25.0%), 30s (25.0%), 40s (25.0%), and 50s or older (25.0%). Educational attainment was relatively high: 71.0% of participants (*N* = 213) held a bachelor’s degree, 12.7% (*N* = 38) had completed high school, 4% (*N* = 12) are currently enrolled in undergraduate school, and 12.3% (*N* = 37) had either completed or were currently enrolled in graduate programs.

All respondents reported having watched the entire series, as confirmed through the screening process. Regarding viewing timing, 41.7% (*N* = 125) watched the series within 2 weeks of its initial release between March 28 and April 10, 2025, 34.3% (*N* = 103) completed it within one to 2 months after release (April–May 2025), and 23.3% (*N* = 70) reported binge-watching multiple episodes after June 2025. This distribution indicates that the sample included both early and later viewers following the initial release.

### 2.3 Measures

All constructs were measured using multi-item scales adapted from established instruments in prior narrative persuasion research. Responses were recorded on a 5-point Likert scale ranging from 1 (“strongly disagree”) to 5 (“strongly agree”). All reliability coefficients exceeded 0.70, indicating acceptable internal consistency.

#### 2.3.1 Character identification

It refers to the process through which viewers experience a character’s emotions, goals, and perspectives as their own (Cohen, 2001; Tal-Or and Cohen, 2010). It involves both emotional empathy and cognitive perspective sharing. Three items were adapted from Tal-Or and Cohen (2010) and Busselle and Bilandzic (2009). Participants were instructed to respond with reference to the primary protagonist of the series. Sample items included: “I empathized with the character’s emotions and experiences,” “I felt as if I were experiencing the character’s situation from his or her perspective,” and “I understood the character’s attitude toward life.” Cronbach’s *α* was 0.80.

#### 2.3.2 Perceived verisimilitude

Perceived verisimilitude assesses the degree to which the narrative is perceived as credible and logically coherent within its story world (Busselle and Bilandzic, 2009; Van Laer et al., 2014). This work prioritizes narrative credibility within its story world. Two items were used, including: “The events in the drama felt plausible within the narrative world,” “The storyline was internally coherent and made sense within the story context.” Cronbach’s *α* was 0.79.

#### 2.3.3 Cultural familiarity

Cultural familiarity assesses the degree to which viewers recognized and could readily interpret the cultural elements depicted in the drama. In this study, this construct is treated as a contextual antecedent of narrative transportation, reflecting how preexisting cultural knowledge and lived experience may support entry into the narrative world (Green, 2004; Van Laer et al., 2014). Two items assessed familiarity with cultural expressions, such as: “The culture and lifestyle portrayed in the drama felt familiar to me,” and “I could naturally relate to the dialects, customs, or foods shown in the drama.” Cronbach’s *α* was 0.71.

#### 2.3.4 Narrative transportation

Narrative transportation represents the overall experience of being mentally and emotionally absorbed into a story world (Green and Brock, 2000). Five items from Green and Brock (2000) were adapted for long-form audiovisual content. Sample items included: “I felt as if I was temporarily separated from reality while watching the drama,” “I became completely absorbed in the atmosphere of the story,” and “At times, it felt as though I was inside a scene of the drama.” Cronbach’s *α* was 0.87.

#### 2.3.5 Bittersweet emotion

Bittersweet emotion represents a mixed emotional state in which warmth and sadness coexist, often accompanied by moral reflection (Hanich et al., 2014; Newman and Sachs, 2023; Oliver and Hartmann, 2010). In the present study, the items were intended to capture a bittersweet-leaning mixed emotional response centered on warmth and sadness. This usage follows prior work on bittersweetness as an ambivalent affective state (Vaccaro et al., 2020) and evidence that happiness and sadness can co-occur in bittersweet moments (Larsen and Green, 2013). Accordingly, the construct was used to index a specific mixed-feeling pattern rather than a broad category of meaningful emotional reactions. Five items measured experiences such as being moved, nostalgic reflection, and emotional depth. Sample items included: “I felt moved while watching the story,” “The drama made me feel nostalgic about past relationships or moments,” and “Certain scenes made me feel both heartache and warmth at the same time.” Cronbach’s *α* was 0.89.

#### 2.3.6 Relational moral beliefs

Relational moral beliefs refer to post-viewing evaluative endorsement of relational norms emphasizing empathy, fairness, and relational equality. Rather than measuring belief change directly across time, the present study assesses participants’ conscious agreement with belief statements aligned with relational ethics after watching the drama. This construct captures normative endorsement rather than readiness for action. Four items captured the extent to which respondents endorsed such relational orientations. Sample items included: “After watching the drama, I believe that empathy should guide family and community relationships,” “After watching the drama, I believe that fairness should take precedence over rigid hierarchical authority in relationships,” “I consider mutual respect to be essential in close relationships,” “I believe that relational equality strengthens long-term social bonds.” Cronbach’s *α* was 0.83.

#### 2.3.7 Relational moral behavioral intentions

Relational moral behavioral intentions refer to one’s readiness to express relational and prosocial values in everyday action. Unlike relational moral beliefs, which assess evaluative agreement with relational norms, this construct focuses on participants’ self-reported readiness to enact those norms in future interpersonal situations. Drawing on narrative persuasion and social learning perspectives (Slater and Rouner, 2002; Green and Appel, 2024), four items measured intentions related to communication, mutual care, and openness to difference. Sample items included: “After watching the drama, I plan to practice greater empathy in family or community interactions,” “I intend to prioritize fairness in my personal relationships,” “I intend to address unequal relational norms in my everyday interactions when appropriate,” and “I intend to act in ways that reflect mutual respect in my interpersonal relationships.” Cronbach’s *α* was 0.90.

## 3 Results

### 3.1 Descriptive statistics and bivariate correlations

Prior to evaluating the measurement and structural models, descriptive statistics and bivariate correlations among the main study variables were examined. As shown in Table 1, all focal variables were positively correlated in directions consistent with the proposed model. In particular, narrative transportation showed relatively strong positive correlations with bittersweet emotion and relational moral beliefs, and relational moral beliefs were strongly correlated with relational moral behavioral intentions.

**Table 1:** Means, standard deviations, and correlations among the main study variables.

Variable	*M*	SD	1	2	3	4	5	6	7
1. Character identification	4.28	0.59	—						
2. Perceived verisimilitude	4.26	0.69	0.63^**^	—					
3. Cultural familiarity	3.89	0.73	0.40^**^	0.37^**^	—				
4. Narrative transportation	4.05	0.69	0.67^**^	0.64^**^	0.43^**^	—			
5. Bittersweet emotion	4.37	0.69	0.65^**^	0.63^**^	0.32^**^	0.71^**^	—		
6. Relational moral beliefs	3.85	0.73	0.46^**^	0.44^**^	0.35^**^	0.63^**^	0.53^**^	—	
7. Relational moral behavioral intentions	3.42	0.88	0.25^**^	0.29^**^	0.35^**^	0.50^**^	0.31^**^	0.76^**^	—

**p <0.01.

### 3.2 Measurement model assessment

A CFA was conducted to assess the measurement model. The hypothesized model was estimated using SEM with maximum likelihood estimation in AMOS. The measurement model showed an acceptable fit: *χ*^2^(265) = 727.11, *χ*^2^/*df* = 2.74, RMR = 0.06, CFI = 0.90, NFI = 0.86, TLI = 0.89, IFI = 0.91, and RMSEA = 0.08, 90% CI [0.070, 0.083]; the model estimated 60 free parameters, and Hoelter’s critical N values were 125 at the 0.05 level and 133 at the 0.01 level. Relative to the independence model, the hypothesized model showed substantial incremental improvement. The saturated model was treated as a statistical benchmark rather than as a substantive theoretical alternative (Table 2).

**Table 2:** Confirmatory factor analysis results.

Construct	Items	beta	*β*	SE	*t*	AVE	CR
Character identification	Q1	1.00	0.75			0.57	0.80
	Q2	0.99	0.78	0.08	12.67		
	Q3	0.95	0.73	0.08	11.93		
Perceived verisimilitude	Q4	1.00	0.74			0.66	0.80
	Q5	1.34	0.88	0.10	13.07		
Cultural familiarity	Q6	1.00	0.73			0.56	0.71
	Q7	1.19	0.76	0.16	7.55		
Narrative transportation	Q8	1.00	0.70			0.58	0.87
	Q9	1.09	0.80	0.08	12.94		
	Q10	0.94	0.80	0.07	13.01		
	Q11	1.09	0.73	0.09	11.85		
	Q12	1.07	0.75	0.09	12.23		
Bittersweet emotion	Q13	1.00	0.78			0.63	0.89
	Q14	1.08	0.80	0.07	14.96		
	Q15	1.05	0.79	0.07	14.53		
	Q16	1.09	0.77	0.07	14.21		
	Q17	1.06	0.82	0.07	15.29		
Relational moral beliefs	Q18	1.00	0.71			0.57	0.84
	Q19	0.99	0.70	0.09	11.34		
	Q20	1.05	0.83	0.08	13.17		
	Q21	0.94	0.77	0.08	12.34		
Relational moral behavioral intentions	Q22	1.00	0.80			0.70	0.90
	Q23	1.07	0.85	0.07	16.28		
	Q24	1.10	0.82	0.07	15.65		
	Q25	1.19	0.87	0.07	16.96		

All standardized factor loadings met or exceeded the 0.70 threshold (e.g., identification: 0.73–0.78; verisimilitude: 0.74–0.88; familiarity: 0.73–0.76; transportation: 0.70–0.80; emotion: 0.77–0.82; beliefs: 0.70–0.83; behaviors: 0.80–0.87), supporting convergent validity (Anderson and Gerbing, 1988). Construct reliability (CR) values were all above 0.70, and average variance extracted (AVE) values exceeded 0.50, further indicating adequate convergent validity. Discriminant validity was established as the square root of each construct’s AVE exceeded the inter-construct correlations (Fornell and Larcker, 1981).

### 3.3 Structural model results

The structural model demonstrated statistically significant associations among the proposed variables. Character identification was positively associated with narrative transportation (*β* = 0.48, *p* < 0.001), consistent with H1a. Perceived verisimilitude also showed a significant association with narrative transportation (*β* = 0.36, *p* < 0.001), consistent with H1b. Cultural familiarity showed a smaller yet statistically significant association with transportation (*β* = 0.13, *p* = 0.03), consistent with H1c. Narrative transportation was positively associated with bittersweet emotion (*β* = 0.91, *p* < 0.001), consistent with H2. Transportation was also significantly associated with relational moral beliefs (*β* = 0.38, *p* = 0.02), consistent with H3, and bittersweet emotion showed a direct association with relational moral beliefs (*β* = 0.36, *p* = 0.03), consistent with H4. Finally, relational moral beliefs were positively associated with relational moral behavioral intentions (*β* = 0.80, *p* < 0.001), consistent with H6.

**Figure 2:**
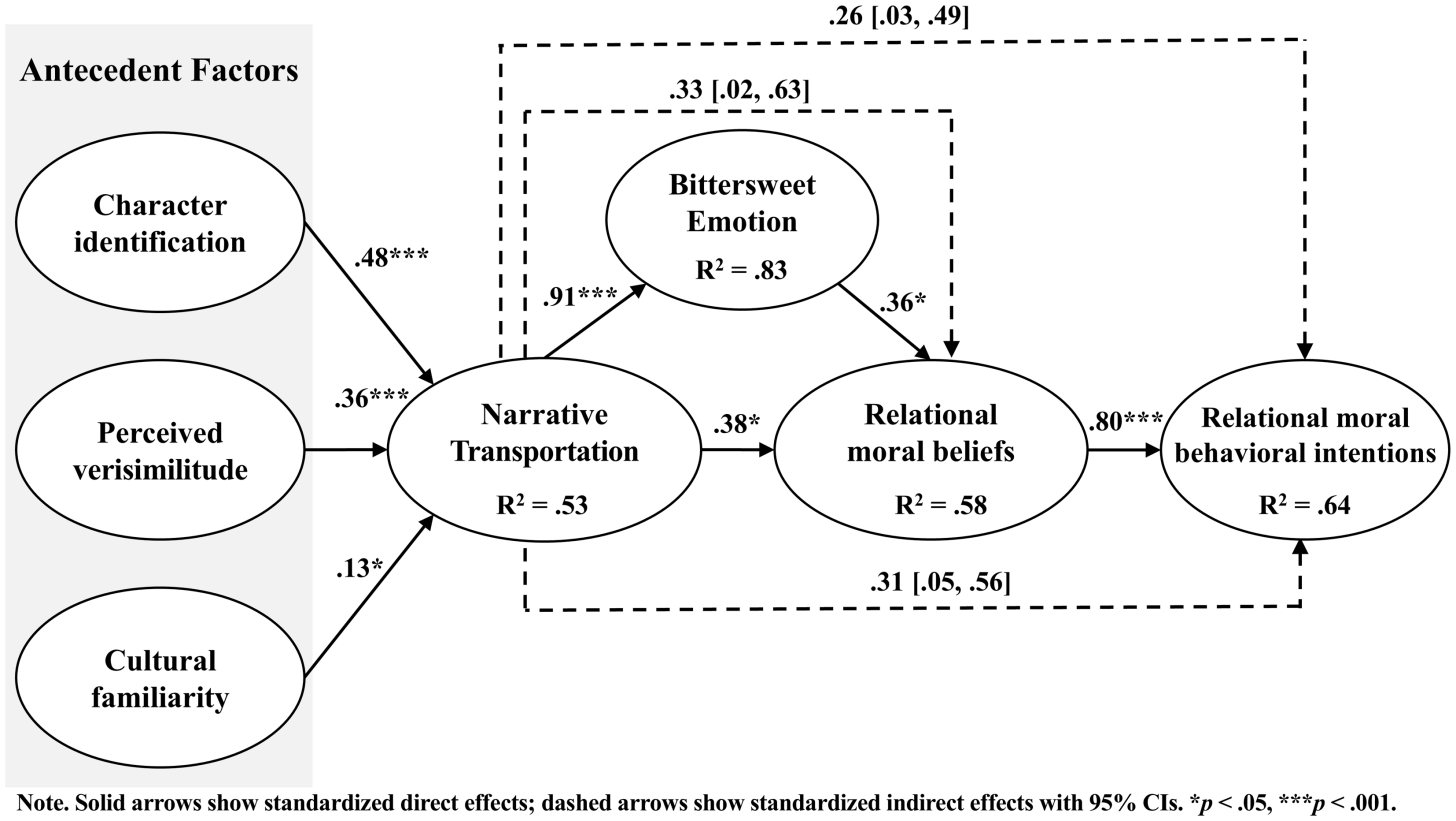
Structural model with standardized path coefficients.

Table 3 presents the standardized path coefficients and significance levels for the hypothesized relationships. Figure 2 illustrates the structural model, depicting the associations among narrative antecedents, transportation, bittersweet emotional responses, relational moral beliefs, and behavioral intentions.

**Table 3:** Path analysis results of SEM.

IVs	DVs	*β*	SE	*t*
Character identification	Narrative transportation	0.48	0.12	4.57^***^
Perceived verisimilitude	Narrative transportation	0.36	0.11	3.70^***^
Cultural familiarity	Narrative transportation	0.13	0.06	2.21^*^
Narrative transportation	Bittersweet emotion	0.91	0.07	11.79^***^
Narrative transportation	Relational moral beliefs	0.38	0.19	2.28^*^
Bittersweet emotion	Relational moral beliefs	0.36	0.20	2.15^*^
Relational moral beliefs	Relational moral behavioral intentions	0.80	0.08	10.84^***^

*p <0.05; **p <0.01; ***p <0.001.

### 3.4 Mediation and indirect effects

Indirect effects were tested using bootstrapping procedures with 5,000 bootstrap samples and a 95% bias-corrected confidence interval. As shown in Table 4, narrative transportation had a significant indirect association with relational moral beliefs via bittersweet emotion (*β* = 0.33, 95% CI [0.02, 0.63]), indicating partial mediation and consistent with H5. The indirect pathway from narrative transportation to relational moral behavioral intentions through relational moral beliefs was also significant (*β* = 0.31, 95% CI [0.05, 0.56]), consistent with H7. In addition, the serial indirect association from narrative transportation to relational moral behavioral intentions via bittersweet emotion and relational moral beliefs was significant (*β* = 0.26, 95% CI [0.03, 0.49]), consistent with H8. In the present study, these indirect effects are interpreted as model-consistent associations rather than as evidence of causal mediation or temporally ordered psychological processes.

**Table 4:** Mediation path analysis results.

Path	Direct effect	Indirect effect	SE	CI (95%) [LL, UL]	Mediation type
Narrative transportation → Bittersweet emotion → Relational moral beliefs	0.38	0.33	0.16	[0.02, 0.63]	Partial
Narrative transportation → Relational moral beliefs → Relational moral behavioral intentions	n/a	0.31	0.13	[0.05, 0.56]	Full
Narrative transportation → Bittersweet emotion → Relational moral beliefs → Relational moral behavioral intentions	n/a	0.26	0.12	[0.03, 0.49]	Serial

### 3.5 Total effects and explained variance

Narrative transportation showed a strong direct association with bittersweet emotion (*β* = 0.91, *p* < 0.001) and a moderate total association with relational moral beliefs (β_total = 0.71), comprising both a direct association (β_direct = 0.38, *p* = 0.02) and an indirect association via bittersweet emotion (β_indirect = 0.33, 95% CI [0.02, 0.63]).

The total association between narrative transportation and relational moral behavioral intentions was also substantial (β_total = 0.57, 95% CI [0.41, 0.73]). This association was mainly indirect, operating through two interrelated pathways: a cognitive-belief pathway (Narrative transportation → Relational moral beliefs → Relational moral behavioral intentions, β_indirect = 0.31, 95% CI [0.05, 0.56]) and a serial emotional-cognitive pathway (Narrative transportation → Bittersweet emotion → Relational moral beliefs → Relational moral behavioral intentions, β_indirect = 0.26, 95% CI [0.03, 0.49]).

The antecedent variables jointly accounted for 53% of the variance in narrative transportation (*R*^2^ = 0.53). Narrative transportation accounted for 83% of the variance in bittersweet emotion (*R*^2^ = 0.83). Together, narrative transportation and bittersweet emotion accounted for 58% of the variance in relational moral beliefs (*R*^2^ = 0.58). Relational moral beliefs accounted for 64% of the variance in relational moral behavioral intentions (*R*^2^ = 0.64).

## 4 Discussion

The present findings indicate that persuasive engagement with serialized streaming narratives may involve the joint operation of interpretive processing and emotionally reflective responses. In this respect, the applicability of the ETIM can be extended to narrative formats characterized by sustained exposure across multiple episodes. Previous research has frequently conceptualized narrative persuasion as emerging from relatively brief encounters with story content, in which emotional absorption and reduced counterarguing facilitate belief-oriented processing (Green and Brock, 2000; Slater and Rouner, 2002). At the same time, responses related to moral evaluation have been discussed in relation to viewers’ reflective engagement with meaningful narrative experiences, particularly when emotional reactions such as being moved or elevated prompt consideration of interpersonal values and social connectedness (Nabi and Green, 2015; Oliver et al., 2012). Meaning-oriented engagement with narrative entertainment has been theorized to involve reflective appraisal of values and social connectedness beyond immediate affective enjoyment (Oliver and Raney, 2011).

These findings should be interpreted as cross-sectional associations rather than evidence of causal influence. Because participants reported on their viewing experiences retrospectively after completing the series, the present design cannot determine whether transportation temporally preceded bittersweet emotion, whether beliefs developed during viewing, or whether these variables were reconstructed together in memory at the time of the survey.

The observed results further suggest that transportation may be associated not only with immediate emotional immersion but also with interpretive processes that accumulate across extended viewing. Repeated encounters with evolving interpersonal dilemmas may provide viewers with opportunities to reconsider narrative meaning in light of subsequent relational outcomes. Research on streaming consumption has indicated that such repetition may reinforce imagery and deepen character-based attachment over time (Warren, 2020), while binge-viewing patterns have been linked to increased transportation and parasocial continuity across episodes (Erickson et al., 2019). Binge-viewing patterns have been associated with increased transportation and parasocial continuity across episodes (Pittman and Sheehan, 2015). These characteristics position serialized streaming dramas as suitable contexts for examining transportation as an ongoing experiential process embedded in naturalistic media use.

The association between transportation and bittersweet emotional responses is compatible with the ETIM assumption that emotionally mixed reactions may accompany immersive narrative engagement. The strong association between transportation and bittersweet emotion is consistent with the view that immersive narrative engagement may be accompanied by an integrated mixed-feeling state, especially in contexts where happiness and sadness are experienced together rather than sequentially (Larsen and Green, 2013; Vaccaro et al., 2020). In the present study, however, bittersweet emotion is interpreted not as a synonym for transportation itself, but as a more specific ambivalent affective response that may emerge within transportation and help explain viewers’ reflective openness to relational meanings. At the same time, the very strong association between transportation and bittersweet emotion suggests that these constructs may be closely aligned at the empirical level in this sample, even though they are treated as conceptually distinct in the present study. This pattern may reflect the extent to which immersive engagement and emotionally mixed response co-occur in long-form narrative experience. Although discriminant validity met conventional criteria in the measurement model, the magnitude of this association suggests that the empirical proximity between the two constructs should be acknowledged when interpreting the findings. Rather than reflecting transient mood states, these responses may provide affective contexts in which viewers reconsider relational norms depicted within the story world. Emotionally mixed responses to narrative entertainment have previously been described as facilitating integrative meaning-making through the joint involvement of affective and cognitive appraisal processes (Bartsch and Oliver, 2011). The serial indirect pathway identified in the model suggests that emotionally reflective responses may be associated with subsequent endorsement of relational moral beliefs, which in turn relate to behavioral intentions oriented toward interpersonal consideration. This pattern corresponds with prior conceptualizations of narrative persuasion as involving both experiential simulation and downstream evaluative processing (Van Laer et al., 2014; Green and Appel, 2024).

Variance estimates from the model indicate that both cognitive absorption and emotionally mixed engagement may contribute to relational belief-oriented outcomes in streaming contexts. Character identification, perceived realism, and cultural familiarity jointly explained a substantial proportion of variance in transportation, suggesting that narrative immersion remains contingent upon interpretable social cues and coherent character motivation. The relatively modest association of cultural familiarity with transportation is consistent with our treatment of this construct as contextual antecedent that supports narrative entry, rather than as a primary persuasive mechanism in itself. Transportation was also associated with bittersweet emotional responses, and the combination of these processes accounted for a considerable proportion of variance in relational moral beliefs. These findings are compatible with a dual-route interpretation in which transportation relates to endorsement of relational moral beliefs both directly through interpretive simulation and indirectly through emotionally reflective engagement. A similar point applies to the strong association between relational moral beliefs and relational moral behavioral intentions. Although the two constructs are conceptually distinguished in the present study as evaluative endorsement versus self-reported readiness for enactment, their close empirical relationship suggests that belief endorsement and intended action were tightly linked in this sample. This pattern is theoretically plausible, but it should be acknowledged when interpreting the strength of the indirect associations leading to behavioral intentions.

In practical narrative contexts, these findings suggest that emotionally layered storytelling may be relevant to the formation of relational orientations beyond the narrative environment. Rather than depicting overt ideological conflict, serialized streaming narratives often present incremental adjustments in interpersonal expectations through routine social interaction. Such portrayals may provide viewers with interpretive material that becomes cognitively or affectively salient through repeated exposure. Streaming dramas may therefore function not merely as sources of entertainment but as narrative environments in which viewers evaluate interpersonal norms across evolving relational contexts.

Several limitations warrant attention when interpreting the present findings. Although the proposed framework conceptualizes persuasion in serialized streaming as a temporally extended process, the cross-sectional nature of the survey design constrains examination of how transportation and emotionally mixed responses develop across episodes. The serial association observed in the model may reflect cumulative interpretive engagement that emerges over repeated viewing; however, without longitudinal or episode-level assessments, it remains unclear whether these responses arise gradually during exposure or are reconstructed retrospectively following narrative completion. Accordingly, the observed indirect associations should not be interpreted as evidence of causal mediation, but rather as model-consistent patterns in cross-sectional self-report data. Future research employing time-sensitive approaches, such as experience sampling or episode-level measurement, may provide more direct insight into the dynamic interplay between transportation and affective engagement in streaming environments.

The reliance on post-viewing self-report measures also introduces the possibility that participants’ responses were influenced by recall processes rather than moment-to-moment emotional reactions. This concern may be particularly relevant for viewers who completed the series several months prior to participation. Given that emotionally mixed responses are likely to fluctuate across narrative developments, subsequent work could incorporate real-time behavioral indicators or physiological measures of engagement to complement retrospective evaluations. Such approaches may offer a more detailed account of how cognitive and emotional responses evolve during sustained narrative exposure.

## 5 Conclusion

In conclusion, the present study applies the ETIM to the context of serialized streaming narratives by examining how immersive engagement and emotionally mixed responses are associated with relational belief-oriented outcomes. The findings indicate that narrative transportation and bittersweet emotional experience may jointly relate to viewers’ endorsement of interpersonal values and corresponding behavioral intentions within everyday relational contexts.

By considering both interpretive simulation and emotionally reflective engagement, this research contributes to ongoing discussions in narrative persuasion regarding how sustained narrative exposure may be associated with belief-oriented processing beyond immediate affective immersion. More broadly, the results highlight the potential relevance of emotionally complex storytelling as a context in which viewers may engage with interpersonal norms and relational meaning in contemporary digital media environments.

## Data Availability

The datasets generated or analyzed in the current study are not publicly available due to privacy and ethical considerations related to the participants. Access to the data may be granted by the corresponding author upon reasonable request, subject to institutional and ethical approval.
